# Effectiveness of the 10-valent pneumococcal conjugate vaccine on pediatric pneumonia confirmed by ultrasound: a matched case–control study

**DOI:** 10.1186/s12931-022-02115-5

**Published:** 2022-08-01

**Authors:** William Checkley, Shakir Hossen, Eric D. McCollum, Farhan Pervaiz, Catherine H. Miele, Miguel A. Chavez, Lawrence H. Moulton, Nicole Simmons, Arunangshu D. Roy, Nabidul H. Chowdhury, Salahuddin Ahmed, Nazma Begum, Abdul Quaiyum, Mathuram Santosham, Abdullah H. Baqui

**Affiliations:** 1 Division of Pulmonary and Critical Care, School of Medicine, Johns Hopkins University, 1830 E. Monument St, Room 555, Baltimore, MD 21287 USA; 2 Department of International Health, Bloomberg School of Public Health, Johns Hopkins University, Baltimore, USA; 3 Department of Biostatistics, Bloomberg School of Public Health, Johns Hopkins University, Baltimore, USA; 4 Department of Pediatrics, School of Medicine, Johns Hopkins University, Baltimore, USA; 5 Johns Hopkins University –Bangladesh, Dhaka, Bangladesh

**Keywords:** Child health, Pneumonia, Vaccine effectiveness, South Asia, Pneumococcal conjugate vaccine, Ultrasound

## Abstract

**Background:**

Bangladesh introduced the 10-valent pneumococcal conjugate vaccine (PCV10) for children aged < 1 year in March 2015. Previous vaccine effectiveness (VE) studies for pneumonia have used invasive pneumococcal disease or chest X-rays. None have used ultrasound. We sought to determine the VE of PCV10 against sonographically-confirmed pneumonia in three subdistrict health complexes in Bangladesh.

**Methods:**

We conducted a matched case–control study between July 2015 and September 2017 in three subdistricts of Sylhet, Bangladesh. Cases were vaccine-eligible children aged 3–35 months with sonographically-confirmed pneumonia, who were matched with two types of controls by age, sex, week of diagnosis, subdistrict health complex (clinic controls) or distance from subdistrict health complex (community controls) and had an illness unlikely due to *Streptococcus pneumoniae* (clinic controls) or were healthy (community controls). VE was measured using multivariable conditional logistic regression.

**Results:**

We evaluated 8926 children (average age 13.3 months, 58% boys) with clinical pneumonia by ultrasound; 2470 had pneumonia with consolidations ≥ 1 cm; 1893 pneumonia cases were matched with 4238 clinic controls; and 1832 were matched with 3636 community controls. VE increased with the threshold used for consolidation size on ultrasound: the adjusted VE of ≥ 2 doses vs. non-recipients of PCV10 against pneumonia increased from 15.8% (95% CI 1.6–28.0%) for consolidations ≥ 1 cm to 29.6% (12.8–43.2%) for consolidations ≥ 1.5 cm using clinic controls and from 2.7% (− 14.2–17.2%) to 23.5% (4.4–38.8%) using community controls, respectively.

**Conclusions:**

PCV10 was effective at reducing sonographically-confirmed pneumonia in children aged 3–35 months of age when compared to unvaccinated children. VE increased with the threshold used for consolidation size on ultrasound in clinic and community controls alike. This study provides evidence that lung ultrasound is a useful alternative to chest X-ray for case–control studies evaluating the effectiveness of vaccines against pneumonia.

## Introduction

Pneumonia is a leading cause of death in children under five worldwide, with most deaths occurring in sub-Saharan Africa and South Asia [1]. *Haemophilus influenzae* type b (Hib) and *Streptococcus pneumoniae* are the two most common pathogens and historically account for more than 50% of pneumonia deaths [2, 3]. The introduction of Hib and pneumococcal conjugate vaccines over the last two decades have led to important reductions in invasive pediatric disease [3–8].

Measuring the effectiveness of Hib and pneumococcal conjugate vaccines against childhood pneumonia is challenging due to the difficulty in etiological ascertainment which can be caused by various pathogens, singly or in combination [9, 10]. A negative sputum or blood test does not rule out a pulmonary Hib or *S. pneumoniae* infection and lung aspiration, the most definite test [11], is neither practical nor ethical in uncomplicated community acquired pneumonia cases. There is also no gold standard for the diagnosis of pneumonia [9], so vaccine effectiveness (VE) studies typically use a combination of clinical, microbiological, and radiographic criteria as their case definitions [12].

Ultrasound is an available alternative for lung imaging [13–17] but has not yet become a standard clinical tool in general pediatrics. Ultrasound has several advantages over radiography. It is less expensive [15], is portable, has no ionizing radiation, and allows for the quantitative assessment of consolidation size. Here, we describe the role of lung ultrasound for the assessment of pneumonia in a VE study during the rollout of the 10-valent pneumococcal conjugate vaccine (PCV10) in Bangladesh.

## Materials and methods

### Study setting

The Bangladesh Ministry of Health and Family Welfare (MOH&FW) introduced PCV10 in March 2015. Children were scheduled to receive PCV10 at 6, 10, and 18 weeks with catch-up vaccination for children < 12 months. We sought to evaluate the effectiveness of PCV10 against pneumococcal infections in three subdistricts (Zakiganj, Kanaighat, and Beanibazar) of the Sylhet District, Bangladesh [18]. Approximately 770,000 inhabitants live in three subdistricts covering 368 square miles for an average population density of 1500 people/square mile. Each subdistrict has a health complex that offers inpatient and outpatient services. The study population were children aged 3–35 months. A network of community health workers visited homes in the study area every 2 months to promote illness recognition and care seeking.

### Study design

We conducted a matched case–control study to evaluate the impact of PCV10 on pediatric pneumonia between July 5, 2015 and September 30, 2017 [18]. We followed STROBE guidelines for reporting on case–control studies [19]. We first enrolled children aged 3–11 months and increased the upper age limit of enrollment thereafter as previously described [18]. Each case was matched with 2–4 clinic and 2–4 community controls within one-week after diagnosis. Initially, we matched each case to four controls of each type because we were expecting a fewer number of cases and wanted to optimize power [20]. In January 2016 we decided to lowered matching to 2 controls per case due to a higher than anticipated case burden. We will refer to the number of matched cases and controls as matched sets.

### Lung ultrasound

Three experts conducted training and standardization in the use of lung ultrasound for the diagnosis of pediatric pneumonia in 25 general practitioners (GPs) [21]. We used portable ultrasound machines (Edge, Sonosite/Fujifilm, Bothell, WA) with a 13–6 MHz linear array transducer. Children with clinical pneumonia underwent LUS carried out by GPs blinded to radiographic findings. Longitudinal and transverse scans were obtained from six sections: two anterior, two lateral, and two posterior sections [21]. Six-second videos from each section were saved and uploaded to a cloud-based server for panel evaluation. Experts read and provided feedback to local GPs and conducted on-site quality control periodically.

### Screening and case identification

We screened a general pediatric population presenting for sick care at the three subdistrict health complexes. Screening was conducted by one of the 25 study GPs. Parents of children aged 3–35 months who presented with cough or difficulty breathing by self-report or direct observation were asked for consent to participate. Upon consent, children were evaluated for clinical pneumonia, defined as fast breathing (respiratory rate ≥ 50 breaths/minute for children aged 3–11 months and ≥ 40 breaths/minute for those aged 12–35 months) or a clinical sign of respiratory illness (including lower chest wall indrawing, persistent nasal flaring, cyanosis, head nodding or tracheal tugging, grunting, stridor while calm, or crackles or wheeze on chest auscultation), consistent with WHO criteria [22]. Children enrolled into the research study were referred for chest radiography [23] or lung ultrasound. All children with clinical pneumonia were provided treatment.

Bilateral lung ultrasonography was carried out by study GPs blinded to radiographic findings. Ultrasound videos were evaluated by pairs of randomly selected GPs. If the two GPs disagreed on the final diagnosis, an expert acted as a tiebreaker [21]. Unlike chest X-ray [24], there is no standardized definition for what constitutes pneumonia on ultrasound. As a starting point, we defined a sonographically-confirmed pneumonia as a consolidation ≥ 1 cm or pleural effusion and an additional abnormality: consolidation of any size, air bronchogram or ≥ 3 B-lines [13]; however, we found that only 12 of 2482 (0.5% of cases) children with clinical pneumonia had a pleural effusion with an additional abnormality. Given the low frequency of this finding, we limited our analysis to cases with consolidation ≥ 1 cm. GPs measured the size of all consolidations identified and categorized the largest consolidation measured into 0–0.99, 1–1.29, 1.3–1.49, 1.5–1.99, and ≥ 2 cm. If both GPs disagreed on the size range, an expert acted as a tiebreaker.

To be eligible as a case, children with pneumonia had to meet the monthly age window for enrollment [18], have their vaccination status available, and an ultrasound diagnosis within 1 week of presentation. Children with recurrent episodes of pneumonia were excluded.

### Matching

Controls were matched within one month of their age, sex, and either subdistrict health complex for clinic-matched sets or house distance from subdistrict health complex for community-matched sets. Clinic controls were required to have an illness unlikely to be due to *S. pneumoniae*, specifically no respiratory symptoms or signs, no documented fever, no signs of meningitis, nor acute otitis media [18]. Community controls underwent a screening examination to identify any acute signs of an illness consistent with *S. pneumoniae* and caregiver self-reported symptoms in the preceding one week. Healthy children without an illness in the last week were included as community controls [18].

### Assessment of vaccination status

Parents were asked to provide the child’s immunization card and the vaccination dates were recorded. Immunization cards were available in 82% of cases and community controls, and in 85% of clinic controls. If the child’s immunization card was not available, we asked the parents if the child was vaccinated and at which immunization center. Study staff reviewed the immunization center records to confirm the child’s date of vaccination. Children who received ≥ 2 doses of PCV10 at least fourteen days before the date of case identification or control selection were considered as vaccinated [8, 18, 25].

### Biostatistical methods

The primary objective was to assess VE for sonographically-confirmed pneumonia in children aged 3–35 months of age. The sample size to detect an expected VE of 20% was estimated at 1130 cases and 2260 controls (1:2 matching), with 80% power and 95% confidence [18]. We measured VE using odds ratios obtained from conditional logistic regression. We evaluated unadjusted and adjusted VE separately for clinic and community controls for different thresholds of consolidation size (≥ 1 cm, ≥ 1.3 cm, ≥ 1.5 cm and ≥ 2 cm). Adjusted models included covariates listed in Table 1, including measures of socioeconomic status (SES) typical of low- and middle-income countries. An SES score was developed utilizing principal component analysis of house construction materials, infrastructure and household asset variables listed in Table 2. Specifically, the first principal component was used as the SES score for our analyses. We also tested for two-variable interaction effects between ≥ 2 doses of PCV10 and consolidation size (1–1.29, 1.3–1.49, 1.5–1.99, and ≥ 2 cm) and for a linear trend between ≥ 2 doses of PCV10 and consolidation size separately for clinic controls and community controls. Using combined controls to gain power for higher-order interactions, we conducted separate three-variable interaction analyses between ≥ 2 doses of PCV10, consolidation size (1–1.49 or ≥ 1.5 cm) and either: laterality (unilateral or bilateral), age (< 12 and ≥ 12 months) and sex. Since only 0.5% of data were missing, we conducted all analyses with complete data only. We conducted the statistical analyses in R version 4.0.0 [26].

**Table 1:** Demographics, education, household characteristics, location and principal components analysis socioeconomic status score based on house construction materials and infrastructure, and household assets in case and control children enrolled in three subdistricts in Sylhet, Bangladesh (July 2015–September 2017)

	Clinic-matched sets (n = 1893)			Community-matched sets (n = 1832)		
	Case	Clinic control	p-value	Case	Community control	p-value
Demographic variables						
Age in months, mean (SD)	11 (7.2)	11 (7.1)	0.95	11 (7.1)	10.7 (6.9)	0.20
Boys, n (%)	844 (46)	1703 (46)	1.00	876 (46)	1966 (46)	0.96
Maternal education, mean (SD)	5.5 (3.3)	6.3 (3.3)	< 0.001	5.5 (3.3)	6.1 (3.4)	< 0.001
Family, n (%) or mean (SD)						
Family owns the house	1653 (90)	3481 (94)	< 0.001	1707 (90)	3863 (91)	0.24
Father resides at home	1571 (86)	2916 (79)	< 0.001	1624 (86)	3384 (80)	< 0.001
Mother empowered to make decisions	516 (28)	1092 (30)	0.30	551 (29)	1237 (29)	0.97
Mother takes for sick care	755 (41)	1208 (33)	< 0.001	782 (41)	1913 (45)	< 0.01
Uses clean cooking stove and fuel	94 (5)	307 (8)	< 0.001	98 (5)	388 (9)	< 0.001
Number of children under five in household	1.8 (0.8)	1.8 (0.9)	0.58	1.8 (0.9)	1.7 (0.8)	< 0.001
Upazila (Sylhet subdistrict)						
Beanibazar, n (%)	530 (29)	1089 (29)	0.82	543 (29)	1209 (29)	0.79
Zakiganj, n (%)	519 (28)	1020 (28)		528 (28)	1223 (29)	
Kanaighat, n (%)	783 (43)	1587 (43)		822 (43)	1806 (43)	
SES score, mean (SD)	0.4 (1.6)	-0.2 (2)	< 0.001	0.4 (1.6)	0 (1.9)	< 0.001

**Table 2:** House construction materials and infrastructure, household assets, and vaccination status in case and control children enrolled in three subdistricts in Sylhet, Bangladesh (July 2015–September 2017)

	Clinic-matched sets (n = 1893)			Community-matched sets (n = 1832)		
	Case	Clinic control	p-value	Case	Community control	p-value
House construction materials and infrastructure, n (%) or mean (SD)						
Number of rooms	2.5 (1.5)	3 (1.9)	< 0.001	2.5 (1.5)	2.8 (1.8)	< 0.001
Has a thatched roof	1775 (97)	3603 (97)	0.23	1835 (97)	4138 (98)	0.13
Has a mud or clay floor	419 (23)	1318 (36)	< 0.001	432 (23)	1478 (35)	< 0.001
Has a mud wall	1020 (56)	2504 (68)	< 0.001	1055 (56)	2803 (66)	< 0.001
Has piped drinking water	1085 (59)	2360 (64)	< 0.001	1118 (59)	2589 (61)	0.14
Has piped sewer system	597 (33)	1759 (48)	< 0.001	618 (33)	1872 (44)	< 0.001
Has electricity	1415 (77)	3139 (85)	< 0.001	1460 (77)	3520 (83)	< 0.001
Household assets, n (%)						
Has an electric fan	1083 (59)	2607 (71)	< 0.001	1114 (59)	2948 (70)	< 0.001
Has a water pump	113 (6)	466 (13)	< 0.001	113 (6)	466 (11)	< 0.001
Has a CD player	48 (3)	245 (7)	< 0.001	48 (3)	205 (5)	< 0.001
Has a color TV	195 (11)	728 (20)	< 0.001	196 (10)	735 (17)	< 0.001
Has a refrigerator	258 (14)	981 (27)	< 0.001	261 (14)	1040 (25)	< 0.001
Has a cellular telephone	1716 (94)	3528 (95)	< 0.01	1772 (94)	4005 (95)	0.18
Has a computer	24 (1)	134 (4)	< 0.001	25 (1)	112 (3)	< 0.01
Has a washing machine	8 (0)	35 (1)	0.06	8 (0)	46 (1)	0.02
Has a clock	434 (24)	1180 (32)	< 0.001	450 (24)	1217 (29)	< 0.001
Has a sewing machine	89 (5)	228 (6)	0.06	93 (5)	293 (7)	< 0.01
Has a thresher	17 (1)	35 (1)	1.00	18 (1)	50 (1)	0.51
Has a cart	23 (1)	50 (1)	0.86	22 (1)	65 (2)	0.31
Has a bicycle	54 (3)	145 (4)	0.08	57 (3)	205 (5)	< 0.01
Has a car	129 (7)	412 (11)	< 0.001	132 (7)	427 (10)	< 0.001
Has a van	45 (2)	67 (2)	0.13	46 (2)	106 (3)	0.94
Has a bed	1770 (97)	3634 (98)	< 0.001	1827 (97)	4139 (98)	0.01
Has a sofa	252 (14)	961 (26)	< 0.001	258 (14)	980 (23)	< 0.001
Has cabinet(s) in home	1290 (70)	2968 (80)	< 0.001	1337 (71)	3270 (77)	< 0.001
Has domestic or farm animals	1321 (72)	2702 (73)	0.45	1366 (72)	2989 (71)	0.20
Exact number of PCV doses, n (%)						
No dose	519 (27.4%)	1042 (24.6%)	0.02	493 (26.9%)	961 (26.0%)	0.49
One dose	211 (11.1%)	444 (10.5%)	0.46	206 (11.2%)	318 (8.6%)	< 0.01
Two doses	414 (21.9%)	1076 (25.4%)	< 0.01	402 (21.9%)	855 (23.1%)	0.34
Three doses	749 (39.6%)	1676 (39.5%)	1.00	731 (39.9%)	1562 (42.3%)	0.10
Cumulative number of PCV doses, n (%)						
At least one dose	1374 (72.6%)	3196 (75.4%)	0.02	1339 (73.1%)	2735 (74.0%)	0.49
At least two doses	1163 (61.4%)	2752 (64.9%)	< 0.01	1133 (61.8%)	2417 (65.4%)	0.01

### Ethics

We obtained approval from the ethics review boards of icddr,b (PR-13095) in Dhaka, Bangladesh, and the Bloomberg School of Public Health (IRB00005421), Johns Hopkins University in Baltimore, USA.

## Results

### Participant characteristics

**Fig. 1:**
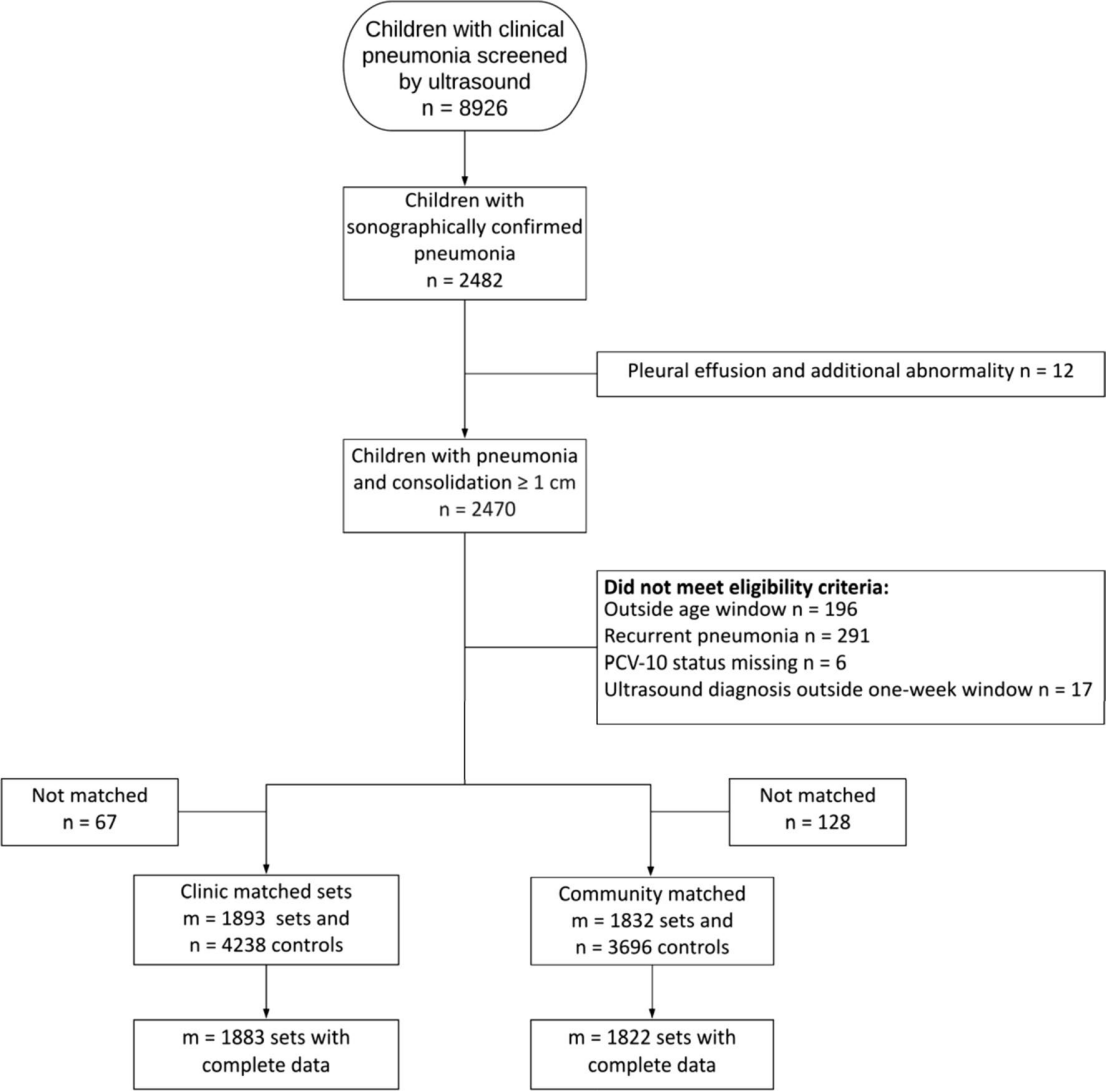
Flow diagram for children screened by ultrasound and those enrolled into the matched case–control study. We include eligibility criteria for cases, the number of matching sets (which is equal to the number of cases) and the number of control children. Only 10 sets (0.5%) had missing data

A total of 8926 children (average age 13.3 months, 58% boys) who met WHO criteria for pneumonia underwent ultrasonography during the study period. Of these, 2482 (27.7%) children met initial criteria for sonographically-confirmed pneumonia: 2470 (99.5%) had a consolidation ≥ 1 cm and 12 (0.5%) had a pleural effusion and additional abnormality. Of the first group, 1706 (69.1%) were consolidations ≥ 1.3 cm, 1327 (53.8%) were ≥ 1.5 cm and 644 (26%) were ≥ 2 cm. Average consolidation size among those ≥ 1 cm was 1.53 cm (SD = 0.50 cm). Children with pneumonia and consolidation ≥ 1 cm were on average younger (12.5 vs. 13.6 months of age; p ≤ 0.001), were more likely to be female (53.6 vs. 59.9%; p ≤ 0.001), had a higher average respiratory rate (52 vs. 50 breaths/minute; p ≤ 0.001), higher average axillary temperature (99.3 vs. 99.1°F; p ≤ 0.001) and lower average oxyhemoglobin saturation (96% vs. 97%; p ≤ 0.001) than children with consolidations < 1 cm. Of the 2470 children with pneumonia and consolidations ≥ 1 cm, 1960 (79.4%) met eligibility criteria for matching (Fig. 1).

### Differences between cases and controls

We matched pneumonia cases to 4238 clinic and 3696 community controls for a total of 1893 clinic-matched sets (97%) and 1832 community-matched sets (93%). Matching by age, sex and subdistrict was successful (Table 1); however, cases were more socioeconomically disadvantaged (Tables 1 and 2). Cases also had a lower proportion of ≥ 2 PCV10 doses than did clinic or community controls (Table 2).

### Vaccine effectiveness against pneumonia

**Fig. 2:**
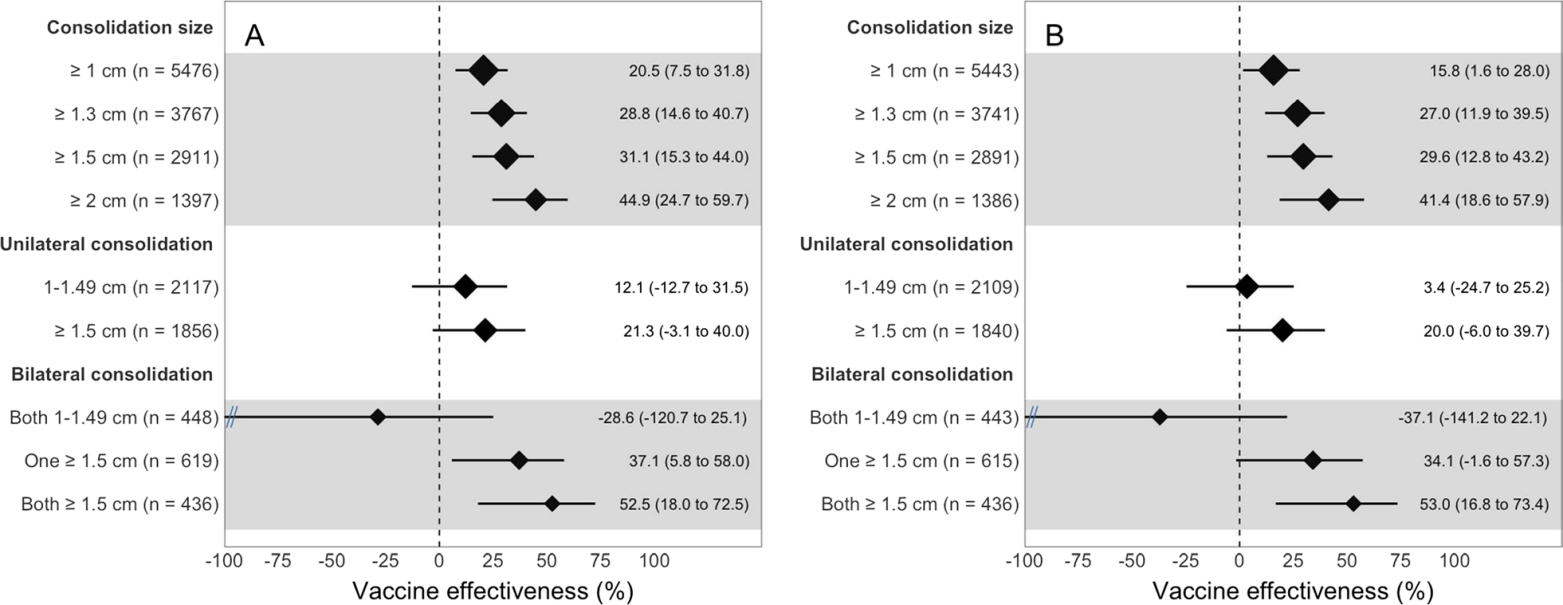
Vaccine effectiveness of receiving at least two doses of the 10-valent pneumococcal conjugate vaccine (PCV10) vs. none against ultrasound-confirmed pneumonia when compared with clinic controls in 3 subdistricts in Sylhet, Bangladesh (July 2015–September 2017). **A** Displays a forest plot of unadjusted vaccine effectiveness of ≥ 2 doses of PCV10 vs. none stratified by consolidation size, laterality, and by age or sex. **B** Displays a forest plot of vaccine effectiveness adjusted for household characteristics, assets, maternal education, whether the father resides in the household, number of children aged under five years living in the household, whether the family owns the house, family owns a clean stove, whether the mother participates in decision making, and propensity to seek care for the child. We stratified cases by consolidation size and, evaluated for interactions between consolidation size (≥ 1.5 or 1–1.49 cm) and laterality. Vaccine effectiveness is represented with a diamond, and the horizontal line is the corresponding 95% confidence interval. The size of the diamond is proportional to the sample size used for each analysis

**Fig. 3:**
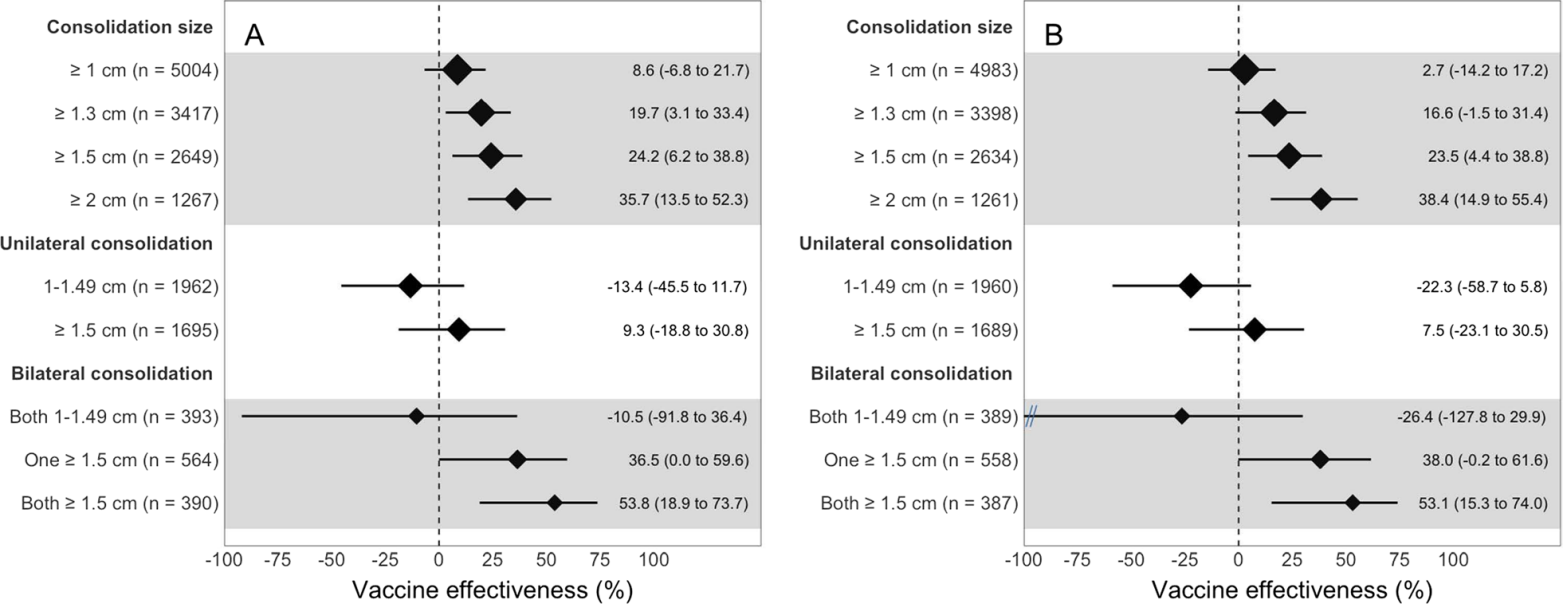
Vaccine effectiveness of receiving at least two doses of the 10-valent pneumococcal conjugate vaccine (PCV10) vs. none against ultrasound-confirmed pneumonia when compared with community controls in 3 subdistricts in Sylhet, Bangladesh (July 2015–September 2017). **A** Displays a forest plot of unadjusted vaccine effectiveness of ≥ 2 doses of PCV10 vs. none stratified by consolidation size, laterality, and by age or sex. **B** Displays a forest plot of vaccine effectiveness adjusted for household characteristics, assets, maternal education, whether the father resides in the household, number of children aged under 5 years living in the household, whether the family owns the house, family owns a clean stove, whether the mother participates in decision making, and propensity to seek care for the child. We stratified cases by consolidation size and, evaluated for interactions between consolidation size (≥ 1.5 or 1–1.49 cm) and laterality (unilateral or bilateral). Vaccine effectiveness is represented with a diamond, and the horizontal line is the corresponding 95% confidence interval. The size of the diamond is proportional to the sample size used for each analysis

We plotted unadjusted and adjusted VEs for ≥ 2 doses of PCV10 vs. non-recipients against pneumonia by consolidation size and laterality for clinic (Fig. 2) and community-matched sets (Fig. 3). VE increased with the threshold used for consolidation size in both clinic- (2 degrees of freedom [df] likelihood ratio test (LRT) p < 0.01 and test for linear trend p = 0.001) and community-matched sets (3 df LRT p = 0.01 and test for linear trend p < 0.001). Adjusted VE increased from 15.8% for consolidations ≥ 1 cm to 29.6% for those ≥ 1.5 cm and to 41.4% for those ≥ 2 cm in clinic-matched sets (Fig. 2). The equivalent increase in community-matched sets was from 2.7%, to 23.5% and 38.4%, respectively (Fig. 3).

Adjusted VEs for ≥ 2 doses of PCV10 vs. non-recipients against pneumonia in either clinic- or community-matched sets were greater for bilateral than unilateral consolidations; however, a larger consolidation size (≥ 1.5 vs. 1–1.49 cm) was a stronger determinant of VE than laterality for both clinic-matched (Fig. 3) and community-matched sets (Fig. 3). When controls were combined, we found that bilateral consolidations had a greater adjusted VE for ≥ 2 doses of PCV10 vs. non-recipients than unilateral consolidations (p = 0.02); however, the overall contribution of laterality was not significant when evaluating the interactions between ≥ 2 doses of PCV10, size and laterality (2 df LRT p = 0.07).

**Fig. 4:**
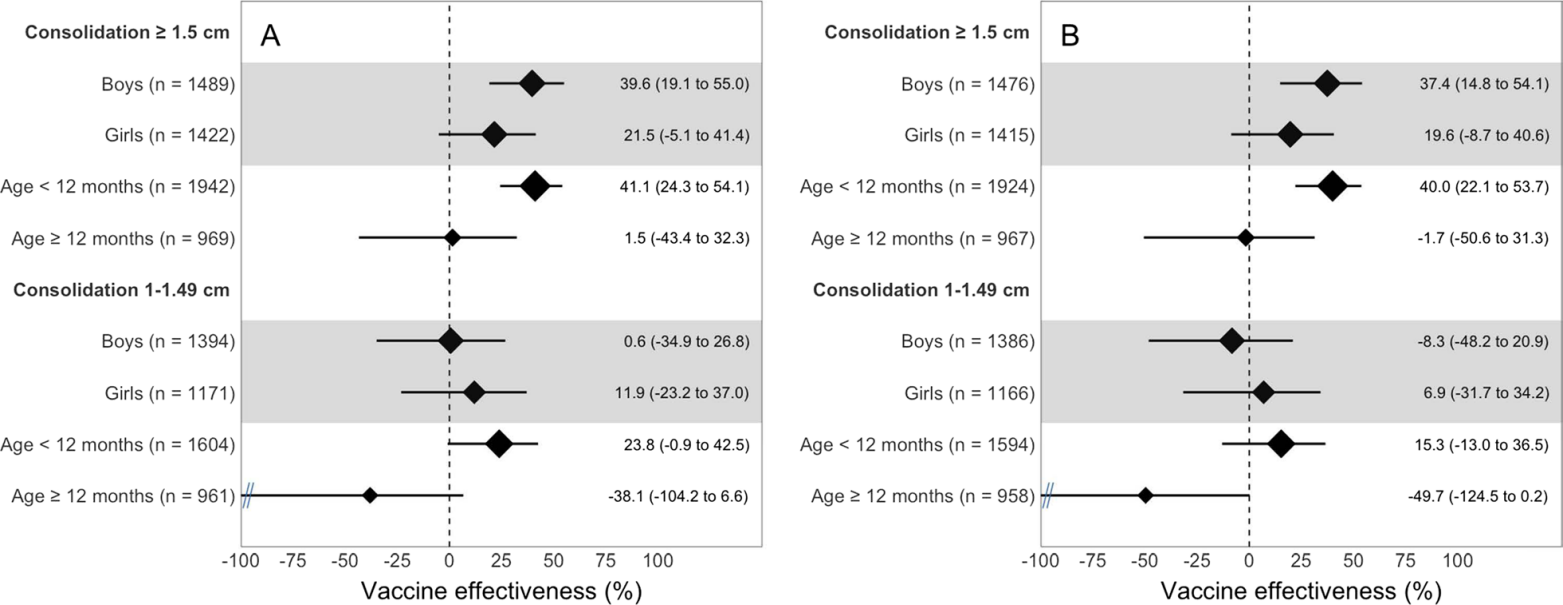
Vaccine effectiveness of receiving at least two doses of the 10-valent pneumococcal conjugate vaccine (PCV10) vs. none against ultrasound-confirmed pneumonia when compared with clinic controls in 3 subdistricts in Sylhet, Bangladesh (June 2015–September 2017). **A** Displays a forest plot of unadjusted vaccine effectiveness of ≥ 2 doses of PCV10 vs. none stratified by consolidation size by age or sex. **B** Displays a forest plot of vaccine effectiveness adjusted for household characteristics, assets, years of schooling for both mother and father, whether the father resides in the household, number of children aged under 5 years living in the household, whether the family owns the house, family owns a clean stove, whether the mother participates in decision making, and propensity to seek care for the child. We stratified cases by consolidation size and, evaluated for interactions between consolidation size (≥ 1.5 or 1–1.49 cm) and age (< 12 or ≥ 12 months) or consolidation size and sex. Vaccine effectiveness is represented with a diamond, and the horizontal line is the corresponding 95% confidence interval. The size of the diamond is proportional to the sample size used for each analysis

**Fig. 5:**
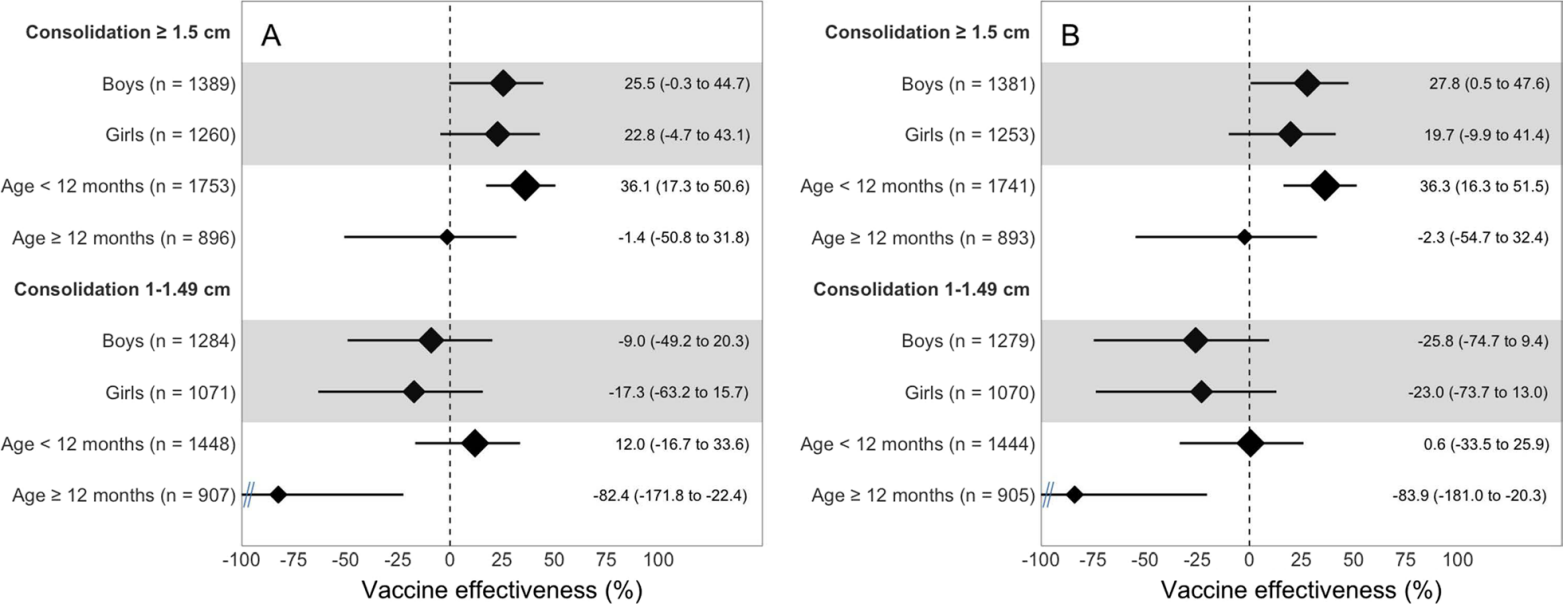
Vaccine effectiveness of receiving at least two doses of the 10-valent pneumococcal conjugate vaccine (PCV10) vs. none against ultrasound-confirmed pneumonia when compared with community controls in 3 subdistricts in Sylhet, Bangladesh (June 2015–September 2017). **A** Displays a forest plot of unadjusted vaccine effectiveness of ≥ 2 doses of PCV10 vs. none stratified by consolidation size by age or sex. **B** Displays a forest plot of vaccine effectiveness adjusted for household characteristics, assets, years of schooling for both mother and father, whether the father resides in the household, number of children aged under 5 years living in the household, whether the family owns the house, family owns a clean stove, whether the mother participates in decision making, and propensity to seek care for the child. We stratified cases by consolidation size and, evaluated for interactions between consolidation size (≥ 1.5 or 1–1.49 cm) and age (< 12 or ≥ 12 months) or consolidation size and sex. Vaccine effectiveness is represented with a diamond, and the horizontal line is the corresponding 95% confidence interval. The size of the diamond is proportional to the sample size used for each analysis

Among those with pneumonia and a consolidation size ≥ 1.5 cm, there were no differences in adjusted VE for ≥ 2 doses of PCV10 vs. non-recipients by sex for clinic-matched (Fig. 4) or community-matched sets (Fig. 5). Indeed, when controls were combined, the adjusted VEs for ≥ 2 doses of PCV10 vs. non-recipients against pneumonia with a consolidation size ≥ 1.5 cm were similar between boys and girls (p = 0.44). Adjusted VE for ≥ 2 doses of PCV10 vs. non-recipients against pneumonia with a consolidation ≥ 1.5 cm was significant in children aged < 12 months in both clinic-matches (Fig. 4) and community-matched sets (Fig. 5) but not in children aged ≥ 12 months. When controls were combined, the adjusted VE for ≥ 2 doses of PCV10 vs. non-recipients against pneumonia with a consolidation ≥ 1.5 cm was greater for children aged < 12 months than for those aged ≥ 12 months (p = 0.05).

## Discussion

We found that PCV10 was effective at reducing sonographically-confirmed pneumonia in children aged 3–35 months of age when compared to unvaccinated children. VE increased with the threshold used for consolidation size on ultrasound in clinic and community controls alike. Specifically, PCV10 prevented about 25% of pediatric pneumonia cases with a consolidation size ≥ 1.5 cm on ultrasound. The vaccine was effective in preventing pneumonia with consolidations ≥ 1.5 cm among younger children but not in older children. Lung ultrasound also has features, like the quantitative assessment of consolidation size, that provides a novel classification for pneumonia studies.

Our group recently reported a lack of VE for ≥ 2 doses of PCV10 vs. non-recipients against radiographically-confirmed pneumonia [23]. There are some notable differences between the radiographic and sonographic studies. First, there was larger number of sonographic cases. Indeed, the sample size was 67% greater than radiographically-confirmed pneumonia cases and 62% greater than the originally planned sample size [18]. Second, sonographically-confirmed cases were better matched to controls when compared to the matching of radiographically-confirmed cases to controls, which may have resulted in less residual confounding and potentially less bias. Third, we used consolidation size as a factor in our analysis. Assessment of consolidation size is not part of the World Health Organization Chest Radiography in Epidemiologic Studies classification [24]. Specifically, we observed a size-response relationship between the threshold used for consolidation and adjusted VE. A larger consolidation size may either be a marker of severity or a more common finding in *S. pneumoniae* infection than in viral infections.

Our analysis agrees with other studies that have found a higher effectiveness of PCV10 against pneumonia in younger than in older children [25, 27, 28]. The significance of this age-related difference is uncertain but may have to do with waning immunity in children aged ≥ 12 months. A third dose provided at a later age as a booster may yield longer lasting immunity against *S. pneumoniae* [27].

Our study has several strengths. First, this is the largest study to conduct lung ultrasound assessment in pediatric pneumonia. Indeed, we conducted over 9,000 assessments in children with suspected pneumonia with an overall yield of 27% positivity for sonographic confirmation. Not only did it afford our analysis with sufficient power for overall and stratified analyses of VE, but it also provided novel information for pneumonia classification such as assessment of consolidation size. Second, we developed a program for standardization and retraining on lung ultrasound for pediatric pneumonia and used a blinded panel for interpretation. A successful training program and panel interpretation is likely responsible for the high-quality data achieved in this study [21]. Third, improved classification of pneumonia using lung ultrasound likely leads to less variability and potentially lower bias. This may help to explain why PCV10 was effective in reducing sonographically-confirmed pneumonia when using both clinic and community controls. Notwithstanding, there are some potential shortcomings in our analysis. We selected an a priori definition for sonographically-confirmed pneumonia that consisted of consolidations ≥ 1 cm or presence of a pleural effusion and an additional abnormality based on our prior work [16, 29] and research from others who suggested that consolidations ≥ 1 cm may be more likely associated with bacterial infections [30, 31]. A more comprehensive analysis that included consolidation size, however, revealed that the original choice of a sonographically-confirmed pneumonia definition may have been too broad and did not consider consolidation size as a factor. A recent study of lung ultrasound in 147 children (median age 42 months) hospitalized with pneumonia in Slovenia found that the median size for the largest consolidation found was 3 and 1.5 cm in bacterial and viral community-acquired pneumonias, respectively [31]. A size-response relationship between consolidation size and VE in both clinic-matched and community-matched sets is reassuring. We recommend that a larger consolidation threshold should be used in VE of PCV10 when using lung ultrasound. Furthermore, the presence of pleural effusion with a consolidation < 1 cm, air bronchogram or ≥ 3 B-lines was a rare finding (< 0.5%) and of unclear significance for pediatric pneumonia.

In summary, we found that PCV10 was effective at reducing community cases of pneumonia with large consolidations (≥ 1.5 cm) on ultrasound in Bangladesh. VE was greater in younger children than in older children. Future studies testing a third dose as a booster at a later age need to be conducted to better understand the significance of age-related immunogenicity. Finally, this study provides evidence that lung ultrasound is a useful alternative to chest X-ray for case–control studies evaluating the effectiveness of vaccines against pneumonia.

## Data Availability

Data and materials can be obtained from the corresponding author upon request.
